# Promoting meaningful activities by occupational therapy in elderly care in Belgium: the ProMOTE intervention

**DOI:** 10.1186/s12877-024-04797-6

**Published:** 2024-03-20

**Authors:** Leen De Coninck, Anja Declercq, Leen Bouckaert, Carola Döpp, Maud J.L. Graff, Bert Aertgeerts

**Affiliations:** 1 Academic Center for General Practice, Department of Public Health and Primary Care, KU Leuven, Kapucijnenvoer 7, Leuven, 3000 Belgium; 2 CEBAM Belgian Center for Evidence-based Medicine vzw, Kapucijnenvoer 7, Leuven, 3000 Belgium; 3 LUCAS Center for Care Research and Consultancy & CESO Center for Sociological Research, KU Leuven, Minderbroedersstraat 8, Leuven, 3000 Belgium; 4 Department of Occupational Therapy, Artevelde University of Applied Sciences, Voetweg 66, Ghent, 9000 Belgium; 5 Scientific Institute for Quality of Health Care, Department of Rehabilitation, Radboudumc Research Institute, Radboud University Medical Center, Houtlaan 4, Nijmegen, 6525 XZ The Netherlands

**Keywords:** Frail older adults, Informal caregivers, Primary care, Functional performance, Social participation, Adherence, Intervention development

## Abstract

**Background:**

Older people want to age in place. Despite advancing functional limitations and their desire of aging in place, they are not always faithful to therapy that maintains independence and promotes safety. Occupational therapists can facilitate aging in place. Occupational therapy is defined as the therapeutic use of everyday life occupations with persons, groups, or populations for the purpose of enhancing or enabling participation.

**Aim:**

To describe the content a high-adherence-to-therapy and evidence-based occupational therapy intervention to optimize functional performance and social participation of home-based physically frail older adults and wellbeing of their informal caregiver, and the research activities undertaken to design this intervention.

**Methods:**

A roadmap was created to develop the occupational therapy intervention. This roadmap is based on the Medical Research Council (MRC) framework and is supplemented with elements of the Intervention Mapping approach. The TIDieR checklist is applied to describe the intervention in detail. A systematic review and two qualitative studies substantiated the content of the intervention scientifically.

**Results:**

The application of the first two phases of the MRC framework resulted in the ProMOTE intervention (Promoting Meaningful activities by Occupational Therapy in Elderly). The ProMOTE intervention is a high-adherence-to-therapy occupational therapy intervention that consists of six steps and describes in detail the evidence-based components that are required to obtain an operational intervention for occupational therapy practice.

**Conclusion:**

This study transparently reflects on the process of a high-quality occupational therapy intervention to optimize the functional performance and social participation of the home-based physically frail older adult and describes the ProMOTE intervention in detail. The ProMOTE intervention contributes to safely aging in place and to maintaining social participation. The designed intervention goes beyond a description of the ‘what’. The added value lies in the interweaving of the ‘why’ and ‘how’. By describing the ‘how’, our study makes the concept of ‘therapeutic use-of-self’ operational throughout the six steps of the occupational therapy intervention. A further rigorous study of the effect of the ProMOTE intervention on adherence, functional performance and social participation is recommended based to facilitate the implementation of this intervention on a national level in Belgium.

**Supplementary Information:**

The online version contains supplementary material available at 10.1186/s12877-024-04797-6.

## Background

68% of the older adult population (65 years and older) is currently living with multiple chronic conditions [1]. Multiple chronic conditions in this population are associated with decreased performance of meaningful daily and social activities. Although, being able to participate in physical and social activities are key determinants of successful and healthy aging [2]. Interventions aiming to improve independence in performing meaningful daily activities and to maintain social participation are an effective way to improve the health and wellbeing of frail older adults. As a result, it also reduces the use of, and the costs associated with, ongoing homecare services [3–5].

Occupational therapists are health professionals whose main goal is to enable engagement in meaningful daily activities within the individual’s living environment, thereby fostering their health and well-being [6]. Occupational therapy (OT) is defined as the therapeutic use of everyday life occupations with persons, groups, or populations for the purpose of enhancing or enabling participation [7].

Skilled in supporting older adults in managing their multiple chronic conditions [8], occupational therapists evaluate the requirements of the elderly and their informal caregivers in a person-centered approach. They facilitate the assumption of responsibility for health by empowering individuals, integrate shared decision-making in goal-setting, implement intervention plans, and collaborate with other healthcare professionals [9, 10] to provide comprehensive support.

Occupational therapists employ evidence-based practices to provide effective care and optimize the utilization of healthcare resources. Evidence-based healthcare is the conscientious, explicit, and judicious use of current best evidence in making decisions about the care of individual patients. This by integrating individual clinical expertise with the best available external clinical evidence from systematic research considering the wishes and values of the individual patient [11].

Previous research on this topic showed that recommendations provided by an occupational therapist have resulted in significant improvement in the older adult’s functional performance, a reduction of falls rate and fewer readmissions to the hospital. These recommendations concern adapting a person’s behavior and if necessary their caregivers’ behaviour, as well as applying assistive devices and home modifications to compensate limited abilities [3, 4, 12–14]. For instance, home making environmental safety adaptations can reduce the number of falls with 26% in people with the age of 60 and older living in the community. Within the older population with higher risk of falling, the reduction of the number of falls increases to 38% [15].

A systematic review on community dwelling older adults indicates that attention should be paid to health promotion. Addressing health promotion, management, and maintenance has been shown to improve occupational performance and quality of life of older adults and is within the scope of OT [16]. Another systematic review shows that occupation-focused and occupation-based intervention using cognitive, behavioral and environmental strategies may significantly improve occupational performance in older, home-dwelling adults with physical health problems. This study also indicates that maintaining achieved improvements is a consistent challenge [17].

A clinical guideline on OT for maintaining the functional performance and social participation of the home based physically frail older adults strongly recommends to involve OT as part of an interprofessional approach in the care of older adults in primary care. This guideline recommends to perform individual home interventions consisting of a combination of actions including increasing health literacy, advising on method change when performing daily operations, advising on aids and practice in their use, advising on home modification including lighting, advising on ergonomics, practicing functional performance, learning self-management strategies, learning fall prevention strategies and advising on service provision [18].

However, the full potential of a recommendation is only achieved when implemented comprehensively. The implementation of specific interventions in practice can be faced with difficulties owing to several factors, one of these being the adherence of the older adults and their caregivers. Despite the desire of the older adults and their caregivers to continue to live independently, therapy adherence is not evident in this population [19, 20]. The causes of (non)adherence to therapy are diverse and include the belief in the effect of an intervention, the involvement of the older adult and their caregivers in goal setting and the degree of interdisciplinary collaboration [20–22].

Because of its properties, such as the number of components involved, uncertainties about adherence and interactions with its context, interventions for multiple chronic conditions are complex interventions which requires flexibility in intervention delivery [23]. Attention should be paid to the resources required to support intervention reach and impact in real world implementation, and to the conditions needed to realize its mechanisms of change. The UK Medical Research Council (MRC) developed a framework for developing and evaluating complex interventions to support researchers to develop complex interventions that consider both the intervention components and its interaction with the context [24]. In addition, there is also the Intervention Mapping approach which is a protocol for developing effective behavior change interventions. Intervention Mapping approach describes the iterative path from problem identification to problem solving or mitigation and consists of six steps [25, 26].

There is a need for a detailed evidence-based intervention that aims to increase the adherence of home-based physically frail older adults and their informal caregiver to OT recommendations for functional performance and social participation. Only if the adherence to therapy is good, the intervention will have full impact.

A growing awareness emphasizes the need for greater transparency in the development of intervention protocols [27]. The appropriateness of the methodologies and rigorousness of the strategies in the development process can be justified by being transparent on the development of the OT intervention protocol. In this way, occupational therapists can demonstrate that their decisions and interventions are grounded in scientific knowledge and research. That is why this study highlights the development and feasibility, as well as the content, of an evidence-based intervention that aims to increase adherence to OT recommendations.

## Aim

To describe the development and feasibility, and content of a high-adherence-to-therapy and evidence-based OT intervention to optimize functional performance and social participation of home-based physically frail older adults and wellbeing of their informal caregiver.

## Method

This study used a mixed method design to develop the evidence-based OT intervention ‘ProMOTE’ (PROmoting Meaningful activities by Occupational Therapy in Elderly).

A roadmap describes in detail the steps taken to develop the ProMOTE intervention and is based on the MRC framework supplemented with elements of the Intervention Mapping approach [24–26]. A previous published meta-analysis and two qualitative studies substantiate the content of the ProMOTE intervention scientifically [9, 28, 29].

The MRC published a framework for researchers on developing and evaluating complex interventions. This framework maximizes the likelihood of developing a successful intervention. Implementing complex interventions should consider the complexity that arises both from the intervention’s components and from its interaction with the context in which it is being implemented. With the framing of the MRC those components are considered [24].

The MRC framework consists of four phases, namely development (phase I), feasibility and piloting (phase II), evaluation (phase III) and implementation (phase IV). Each phase has a common set of core elements in particular considering context, developing and refining program theory, engaging stakeholders, identifying key uncertainties, refining the intervention, and economic considerations. These elements should be considered early and continually revisited throughout the research process, and especially before moving between phases (for example, between feasibility testing and evaluation) [24].

**Fig. 1:**
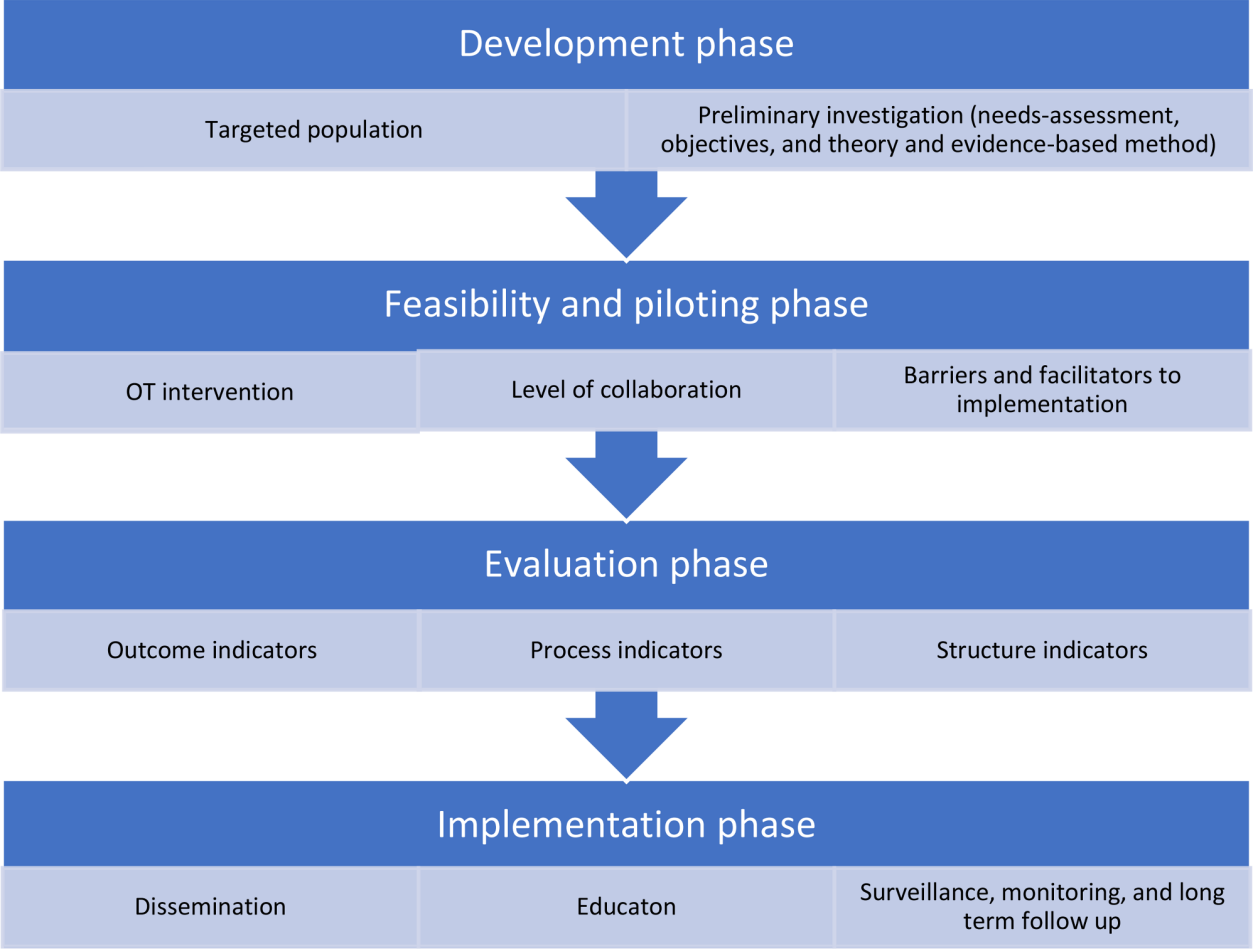
Developmental process of the evidence-based OT intervention based on Intervention Mapping approach incorporated in the phases of the MRC framework

To describe the developmental process in even more depth, components of the Intervention Mapping approach have been added to the MRC framework. The Intervention Mapping approach is anchored in principles that include stakeholders and utilize theories and evidence. The stakeholders must ensure relevance, acceptability, feasibility to implement, and contextual and cultural appropriateness. The Intervention Mapping approach includes six steps, namely assessing needs, identifying intervention objectives and behavioral determinants, designing and/or selecting theory-based methods for behavior change, organizing and producing the intervention, implementing the intervention, and evaluating the intervention [25, 26]. These six steps can be mapped onto the four phases of the MRC framework. (Fig. 1)

The first three steps of the Intervention Mapping approach align with the initial phase of the MRC framework, representing the development stage. These three Intervention Mapping approach steps are the description of a needs-assessment, defining the objectives of the intervention and the description of the theory- and evidence-based methods. Intervention Mapping approach step four concerns integrating methods and the practical applications into the local context, and can be linked to phase II of the MRC framework, i.e. feasibility and piloting. Intervention Mapping approach steps five and six correspond respectively to phases III and IV, evaluation and implementation, of the MRC framework [24–26], which are not the focus of this paper.

The outcome of phases I and II of the roadmap are described in this study and results in the evidence-based OT intervention ProMOTE aimed to increase adherence to treatment, and in recommendations for designing phases III and IV.

In phase I we define the older population and identify determinants of therapy adherence improvement by means of a literature review and qualitative research. The previous published meta-analysis on the effect of an OT intervention on this population and the two qualitative studies, one on the perception of the older adults on their functioning and one on the willingness of professionals to collaborate in primary care, substantiated the ProMOTE intervention scientifically [9, 28, 29].

Phase II consists of piloting the preliminary ProMOTE intervention in one primary care setting, including determining the level of collaboration and detecting the barriers and facilitators of collaboration. Based on the outcome of this pilot, the ultimate evidence-based ProMOTE intervention arises. The TIDieR checklist is used to describe the ProMOTE intervention in detail. This checklist contains the minimum recommended items for describing an intervention. These items are a brief name, the rationale of goals that underpin the intervention, any materials used in the intervention, the processes, activities or procedures the intervention providers carry out, the intervention provider, the modes of delivery of the intervention, where the intervention should be applied, the number of intervention sessions delivered and over what period, level of tailoring the intervention, modifications, attention to adherence and the assessment of the intervention. The TIDieR checklist indicates that additional information can be provided where necessary for the replication of the intervention [27].

Phases III and IV inform on the evaluation and implementation of the OT intervention, which is not the focus here.

## Results

As outlined above, the roadmap for the development of the ProMOTE intervention follows the phases of the MRC framework supplemented with components of the Intervention Mapping approach. The OT intervention emerges as the product of the first two phases. The last two phases serve to inform on the quality measurement of the protocol and the implementation of the OT intervention. The ProMOTE intervention is designed to be implemented in primary care in Belgium.

### Phase I: developing a complex intervention

To develop a complex intervention, problem analysis is a necessary step in identifying which elements must be changed and for whom [24, 26]. The problem in the home-based physically frail older adult population is that adherence to treatment, among which OT interventions, is often weak.

#### Target population

The target population is older adults living at home who are restricted in at least one basic or instrumental activity of daily life and who have experienced one or more of the following in the last 12 months: a fall, restrictions in social participation, receiving home-health services and/or receiving support of an informal care giver. We focus on older adults restricted in at least one activity of daily living because people with problems in functional performance are a the target population of occupational therapists. The age of the target population is 70 and older. Older adults living in a nursing home, or presenting with moderate to severe cognitive impairment or mental health difficulties are not included, notwithstanding that certain aspects of the ProMOTE intervention can also be applied to that population. The reason for not including these populations is the specific approach and/or different context, and because there are already studies on these populations [5, 10, 30].

#### Preliminary investigation (needs assessment, objectives, and method)

The lack of adherence to recommendations or exercises influences the functional performance and social participation of home-based physically frail older adults. Our objective was to develop an evidence-based OT intervention that aims to increase the adherence of this population.

We identified determinants of therapy adherence improvement by means of a literature review and qualitative research [9, 28]. A systematic review with meta-analysis was conducted to assess the effectiveness of OT in improving performance in daily living activities and social participation in community-dwelling physically frail older adults. This study provided convincing evidence that OT improves functioning and social participation in this population and detects the characteristics of effective intervention [28]. Qualitative research was conducted to investigate the perspectives of older adults on their own functioning and social participation, and to detect the factors influencing these components. Assistive devices, the older adult’s dwelling and living environment, professional and informal support and medication are perceived as important external determinants for retaining functioning and social participation. Attitude, social influences, and personal effectiveness emerge as internal factors related to whether a person performs or participates in an activity [9]. Based on the results of these investigations, we developed a preliminary OT intervention for which a pilot implementation was performed to inform on potential barriers [31]. The outcome of this pilot, supplemented with scientific evidence, yielded components for the improvement of therapy adherence which can be assigned to one or more of the following clusters: time to intervention, self-efficacy, prioritization, collaboration, and information exchange. (Table 1)

**Table 1:** Components of adherence improvement based on literature

**Setup of intervention**
- Reducing the time between the initial demand for the intervention and the actual implementation thereof [31, 38] - Building of a trust-relationship [9, 28, 31] - Determining shared decision-based goals [27, 28, 31, 44] - Using strategies to empower older adults, such as improving health literacy [9, 20–22, 28]
**Body of intervention**
- Exploring and discussing barriers and facilitators of an intervention with the older adult [9, 21, 31] - Starting with addressing a limited number of goals that are set as priority by the older adult [15, 31, 49] - Embedding the goals within meaningful daily activities of the older person [9, 21, 28, 31] - Providing objective information on the process of the defined goals [21, 28] - Collaboration with social network [20, 21, 28] - Fluid inter-professional communication and collaboration [21, 28, 31]
**Monitoring and closing of intervention**
- Monitoring the desired changes over a period and providing a follow-up session [21, 31, 38]

### Phase II: piloting and feasibility

#### Piloting the preliminary OT intervention

Piloting the preliminary OT intervention adds value with regards to a sustainable implementation in the broader working field. We piloted the preliminary protocol in a district community center to refine and measure feasibility. One occupational therapist, one final year undergraduate OT student and eight physically frail older adults along with their informal caregivers, if present, participated in the pilot. Interprofessional collaboration was essential due to the complexity of the cases. The content of the OT intervention itself, the level of collaboration and, as mentioned above, the barriers and facilitators to implementation were the subject of measurement [31].

With the pilot implementation, the comprehensiveness and feasibility of the intervention is taken into consideration, and adjustments are made accordingly. The content of the preliminary intervention is refined according to both the participant’s needs and the received feedback. Case finding is conducted prior to ‘functional assessment’. The opportunity to spread the functional assessment over two home visits is suggested if the assessment is too burdensome for the older adult. The step ‘goal setting and developing intervention plan’ has been split due to the specificity and importance of each component. A more detailed description of the adjustments of the intervention has been described elsewhere [31].

#### Determining the level of collaboration

Collaboration is essential in complex cases. In our pilot implementation, the occupational therapist collaborated with the older adult, their informal caregiver, and the involved health professionals. An analysis of the care network confirms that the complex star network reported by Grol (2020) is the most common network type present. This means that the older adult mostly has one informal caregiver and multiple health professionals. In a complex star network, the power in terms of access to information and contacts is held not only by the older adult, but also by another actor, which is often the informal caregiver [32].

Collaboration with the informal caregiver has two main objectives for the occupational therapist: to pursue the older adult’s goals, on the one hand, and to support the informal caregiver themselves to keep the workload bearable, on the other hand.

Each older adult involved in the pilot is receiving treatment from multiple health professionals. During the 10-week period of the pilot, the occupational therapist collaborated with nine other health or well-being professionals or services, specifically social workers, general practitioners, physiotherapists, care coordinators or case managers, home services, a mental health center, a pharmacist, an assistive device store and transport services [31]. In the final version of the evidence-based ProMOTE intervention, particular attention is paid to interprofessional collaboration between these providers.

#### Feasibility: barriers and facilitators of implementation

The success of implementing an intervention that deviates from routine actions is influenced by barriers and facilitators. These influencing factors can be divided into six components: concrete innovation, the individual professional, the user and the informal caregiver, the social context, and the organizational and economic-political context [32]. The innovation level consists of advantages in practice, feasibility, credibility, accessibility, and attractiveness. Determinants of the individual professional include awareness, knowledge, attitude, motivation to change and behavioral routines, while determinants on the level of the user are knowledge, skills, attitude, and compliance. Factors relating to social context include the opinion of colleagues, culture of the social network, collaboration, and leadership. Organizational context refers to organization of care processes, staff, capacities, resources such as computerized decision support systems and structures. Financial arrangements, regulations and policies are determinants of the economic and political context.

We made an inventory of the barriers and facilitators that we detected via various sources, namely scientific evidence, interviews with health professionals and older adults, and insights gained during the pilot. (Table 2) This overview allows us to adapt the preliminary OT intervention to anticipate difficulties with implementation.

**Table 2:** Determinants of success and barriers to the OT intervention

Facilitators	Barriers
**Innovation**	
- Computerized decision support system which detects physically frail older adults or older adults at risk of physical frailty - System that links evidence to the electronic health record (e.g., Evidence Linker) - Secure digital information exchange system - Intervention is evidence based	- Lack of supportive electronic devices
**Individual professional**	
- Health professionals involved receive basic education and training in collaborating with patients with complex chronic conditions - Local health professionals know each other - Health professionals have knowledge of the evidence-based practice method - Methodology for health literacy is part of the basic competences of occupational therapist and other involved health professionals - Freely available scientific evidence translated to local context - Professional pride	- Professionals not believing in the added value of the interventions
**User**	
- Congruence between personal expectations of user(s) and set goals - Insight into the problem, potential cause, and potential consequences for user and, if relevant, informal caregiver - Actions tailored to the pace of user with special attention to actions of assessment phase - Experienced support from peers and family	- Denial of the problem - Lack of motivation - Additional costs of intervention such as the purchasing of assistive devices - Experienced social stigma
**Social context**	
- Congruence between expectations of informal caregiver and set goals - Insight into the problem, potential cause, and potential consequences for informal caregiver - Positive attitude of colleagues towards innovation - Opinion leaders who support the innovation - Collaborative leadership	- Denial of the problem - Lack of motivation or interest of informal caregiver - Content and value of OT in primary care not well known by informal caregivers and health professionals
**Organizational context**	
- Structurally embedded procedure which detects physically frail older adults or high-risk individuals - Integrated care is routine in primary care - OT is already integrated in the daily clinical practice - Innovative intervention implemented within routine actions - Innovative intervention adapted to local context - Indicators (effect-process-structure) available at start of implementation of the intervention	- Ingrained, inflexible organizational system
**Economic and political context**	
- Sufficient allowances for assistive devices and home adaptations - Sufficient compensations for OT intervention	- OT not structurally embedded in primary care in Belgium

#### Ultimate evidence-based ProMOTE intervention

Based on the outcome of previous steps, the preliminary OT intervention has been adjusted. The resulting ProMOTE intervention consists of six components, with collaboration serving as the common thread. The components of this intervention are comparable with the comprehensive geriatric care model and are defined as follow ‘case finding, functional assessment, goal setting, developing an intervention plan, implementing and monitoring the intervention plan and evaluating’ [33]. Collaboration between the older adult, the informal caregiver(s) and the involved health professionals is essential during the intervention. Components important for adherence to the intervention, as described in Table 1, are interwoven during the entire intervention e.g. using strategies to empower the older adult. The intervention is not a strictly linear process, but rather a process in which components are executed iteratively as necessary.

Each step of the intervention describes in detail the goal (why), action (what), approach and communication (how), resources (what and how), collaboration (who and how), timeframe (when) and quality indicator (OI: outcome indicator; PI: process indicator; SI: structure indicator) (Supplement 1).

#### Case finding

Identifying both cases at an early stage and cases at high risk allows for targeted interventions to manage or reduce the risks. Detecting older adults who are physically frail or at risk of physical frailty constitutes part of the daily clinical handling of health professionals, in particular the general practitioner, registered nurse, physiotherapist and occupational therapist, and if relevant also the social worker [31]. Older adults at risk must be referred upon detection to an occupational therapist and by extension to other relevant health professionals. Valuable screening instruments for case finding are the Clinical Frailty Scale of Rockwood, the Frailty Phenotype and BelRAI-screener [34–36].

Decision support systems can support case finding by analyzing complex data, patterns, and patient history. Linking the screening instrument to the computerized decision support system of the electronic health record improves the compliance of the health professional [37]. If no electronic health record with computerized decision support system is available, the health professionals must be empowered to routinely detect older adults at risk. Awareness-raising activities, for instance via information sessions integrated into routine quality meetings, are essential to sustainably maintain compliance of the involved health professional [31].

It may also be necessary to organize interprofessional meetings to get to know the local network of involved health professionals. On the one hand, this helps to become familiarized with each other in the context of referral. On the other hand, it facilitates the acquisition of further knowledge regarding the professional profile of the various care providers. General practitioners indicate that they do not know enough about the possibilities of OT in primary health care. To overcome this barrier in the Belgian context, an evidence-based awareness-raising module ‘OT@home’ has been developed to better familiarize general practitioners with OT. Characteristic of this awareness-raising module is that it can be tailored to the requesting organization [31]. In this way, the intervention meets the requirement for the pursuit of adherence, namely collaboration with involved health professionals.

Interprofessional collaboration does not end with case finding. Interprofessional collaboration remains desirable throughout the entire therapy process, whether or not this takes place under the coordination of a case manager or care coordinator.

#### Functional assessment

The functional assessment is completed for the older adult, and if appropriate, the informal caregiver. To achieve adherence, the first contact between the occupational therapist and the older adult is best continued within a week after the case finding [38].

The occupational therapist uses performance-based measurement instruments to assess the older adult’s ability to perform daily activities and to participate in social life [39]. Within the context of ensuring adherence, evaluating the older adult’s perception of their functional performance and verifying their wishes and needs are just as important as measuring the level of functional performance, social participation and evaluating the home environment [28, 40].

Assessment results may vary depending on the place where the assessment is carried out [41]. That’s why the assessment takes place at the habitat of the older adult. A functional assessment can be exhausting for the older adult, and it may therefore be more advantageous to divide the assessment into two parts to ensure a reliable result [34]. Conducting the assessment in a manner that is tailored to the elderly person and in their habitat contributes towards building a trust-relationship.

In addition to building a trust-relationship, the occupational therapist uses verbal and non-verbal communication strategies. To become an active partner in the therapy process, the health professional, in this case the occupational therapist, pursues proactive commitment from the older adult and their informal caregiver [42, 43]. The occupational therapist therefore not only informs the older adult about the results of the assessment, but also translates the practical consequences thereof for the daily functional performance of the older adult [28, 42].

In consulting the older adult, the occupational therapist exchanges information with relevant health professionals in a secure (digital) way. The involved health professionals, in particular the general practitioner, are important actors in improving adherence [31].

#### Goal setting

Focusing on enhancing health literacy, the occupational therapist addresses the needs of the older adult, and, when applicable, their informal caregiver. The occupational therapist discusses possible care options and treatment opportunities with OT so that the older adult can make informed decisions. The occupational therapist does this shortly after the assessment to further build up the trust-relationship. Rapport between health professionals and the older adult is essential for a good course of therapy [44, 45].

The outcome of the Canadian Occupational Performance Measure (COPM) or another client-centered tool aids in establishing the factors that are important for the older adult. Using principles of Motivational Interviewing supports the health professional in allowing the older adult to find out what their priorities are, to grade the goals and to split the goals up to keep them attainable [31, 45, 46].

The occupational therapist engages with the older adult, and if relevant the informal caregiver, in a participatory model of decision making by collaborating with the person to identify their priorities and goals [31, 47]. Prioritizing at least three of the goals that the older adult identified as high priority contributes to an increase in therapy adherence. When developing the goals, the occupational therapist and the elderly person also place emphasis on feasibility [31, 48, 49].

#### Developing the OT intervention plan

The occupational therapist collaborates with the older adult to determine the optimal OT intervention. In shared decision making, both parties select the actions to undertake in order to achieve the intended goals. The OT intervention focuses on meaningful activities, and consists of a broad spectrum of actions. These include giving information on the intervention opportunities, improving health literacy, skills training, recommending assistive devices and home adaptations, training in the use of the recommended devices and adaptations, recommending and training behavior change, teaching alternative or compensatory strategies, coaching, monitoring and referring.

In cooperation with the older adult, the occupational therapist plans a feasible time schedule to gradually implement these actions, starting with the actions associated with the goals that have been assigned the highest priority.

The general practitioner is an important agent in adherence improvement, and the occupational therapist therefore keeps the general practitioner informed about the OT intervention plan, and if relevant other involved health professionals [31].

#### Implementing the OT intervention plan and monitoring

The developed intervention plan is implemented in the habitat of the older adult. The occupational therapist follows this treatment plan diligently, and the effectiveness of the intervention is closely monitored. However, deviations from the intervention plan are still possible. Based on their therapeutic competences and experiences, the occupational therapist can adjust the intervention plan for well-considered reasons. Any adjustments are done in consultation with the older adult, and if relevant their informal caregiver.

The OT intervention is divided over several sessions. The number of sessions varies and is determined by several factors, such as the complexity of the case, the level of health literacy, the degree of trust or the level of adherence. The occupational therapists communicate about the extent to which a goal has been achieved and about the degree of the older adult’s satisfaction with the therapy process.

Interprofessional meetings are organized where this proves necessary, e.g. to collaborate to increase therapy adherence. These meetings are coordinated by either a case manager or a care coordinator.

#### Evaluating

To measure if the goals of the intervention are achieved, the older adult’s levels of functional performance and social participation are objectively reassessed using performance-based measurement instruments and/or by means of an interview with the older adult regarding their perception of their own functional performance and social participation. Where relevant, the informal caregiver will be involved. A report of the levels of functional performance and social participation is shared with the relevant health professionals.

### Recommendations for phase III and IV

In this section, suggestions for designing phases III and IV are described based on the results of phases I and II.

### Phase III: evaluating interventions and reporting

The evaluation of the ProMOTE intervention should consist of a quality measurement of both the intervention and the OT service.

#### Quality measurement of the intervention

In accordance with the Donabedian model of health quality (1988), quality measures must extend beyond the perspective of the outcomes [49]. Process and structure indicators are equally important because they give insight into the possible reasons for the (lack of) achievement of the treatment effect. Thus, the evaluation must also be conducted from the perspective of structure and process.

Outcome or effect indicators measure changes that occur as a result of the ProMOTE intervention. The effect measurement captures the coverage of the recommendations, referring to the extent to which the recommendation induced effect. The impact of adherence to the recommendations on the level of functional performance, social participation, and quality of life of the older adult, as well as the perceived burden and quality of life of the informal caregiver, are also measured.

Process indicators evaluate how the ProMOTE intervention is delivered and how the older adult deals with the received recommendations. The compliance of the therapist with the intervention should be measured by analyzing their records and registering the extent to which the additional education and supervision sessions are followed. The measurement of how the older adult deals with the recommendation addresses the following subcategories: the content of recommendations and whether recommendations are followed as planned (yes/no). If the recommendations are not followed as indicated, the quantity and coverage will be measured. Quantity consists of the number of recommendations followed, time from when recommendations are followed and if the recommendations are followed for as long as planned.

Structure indicators assess the environmental factors and resources required to deliver a high-quality OT intervention. Contextual factors that might have an impact on the adherence of the older adult to the recommendations can be diverse. Non-adherence to recommendations for safe participation in activities could be due to insufficient allowances for assistive devices and home adaptations. Alongside the impact of contextual factors on adherence of the older adult, the contextual factors that might influence the compliance of the therapist are also measured. Lack of secure digital software such as electronic health records, computerized decision support systems and Evidence Linker might complicate work efficiency.

#### Quality measurement of the OT service

Quality management evaluations of the OT service in general must be conducted continuously. The World Federation of Occupational Therapy (WFOT) supports OTs in conducting quality work by providing the Quality Evaluation Strategy Tool (QUEST). QUEST includes seven dimensions that are most relevant to OT services in their framework. The dimensions include accessibility, appropriateness, effectiveness, efficiency, person-centeredness, safety, and sustainability. In accordance with the Donabedian model of health quality, the seven dimensions are measured by the quality perspectives of structure, process, and outcome [50, 51].

### Phase IV: implementation

To pursue a sustainable implementation of the ProMOTE intervention on a national level, a pilot-implementation study must be conducted. The goal of this pilot study is to select the most effective implementation strategy. Education and inter-vision sessions between peers must be organized to assure and increase compliance of the involved health professionals.

#### Dissemination among professionals

Publishing research is an integral part of an implementation strategy, although the effective application of evidence into practice requires more than the publication of research findings. Platforms that are easily accessible and known by the occupational therapist are valuable tools in dissemination, one example being the website of the national professional association. Within the context of the present study, a clinical guideline has been developed and validated by the national validation committee of the Center for Evidence Based Medicine (CEBAM). The guideline is disseminated via the Belgian Evikey partners, Evidence Based Practicenet (EBPnet.be) and Working group Development of primary care guidelines (EBP-guidelines.be), among others. These platforms disseminate nationally validated guidelines for primary care and are well known by health care professionals in Belgium.

#### Dissemination among older adults and their caregiver

In addition to disseminating among caregivers, it is important that the older adult is also aware of possible answers to their needs. Knowledge translation and dissemination among the older adults is an aspect of implementation that requires attention. This can take multiple forms, for example communications from health care providers or health insurance companies and patient platforms.

Health care providers and insurance companies can inform the older adult individually. They can additionally amplify oral information with flyers, posters, and banners.

National patient platforms, such as Health and Science (Gezondheidenwetenschap.be) for the Dutch speaking part of Belgium or Health information (Infosanté.be) for the French speaking part of Belgium, disseminate reliable information about health in an accessible format. They synthesize and translate the validated Belgian guidelines into lay language and promote them via their website. Elderly advisory and participation bodies of governments can communicate the knowledge in two directions, namely towards the elderly and towards the policy makers. For instance, the Flemish Elderly Council (Vlaamse-ouderenraad.be), which is a non-profit consultation platform consisting of various organizations of and for the elderly, coordinates awareness-raising campaigns to inform the elderly about topics that concern them, and they additionally advise on policy relating to the elderly population.

#### Education

The selection of implementation strategies that consider previously identified barriers to adherence requires careful consideration. Education as a means of implementation is aimed at both higher education institutions and the professional field. It consists of promoting awareness of change, stimulating interest and involvement, creating understanding, developing insight into one’s own routines, developing a positive attitude towards change and creating positive intentions/decisions to change [52]. Educational tools will be developed for both the content of the intervention as well as for the mastering of communication and collaboration skills for the therapists.

#### Surveillance, monitoring, and long term follow up

Integration of the ProMOTE intervention into an organized program facilitates sustainability of the actions into routine. Inter-vision sessions between occupational therapists, preferably with various levels of experience, must ensure that there is an intrinsic motivation to continue monitoring of the intervention. Such inter-vision sessions can be organized regionally and should become part of the regular work of the occupational therapist to support sustainability and quality of treatment.

## Discussion

This article set out to describe the development and feasibility of an evidence-based intervention that aims to increase the adherence of home-based physically frail older adults to OT recommendations for functional performance and social participation and the intervention itself. It also provides recommendations for designing the future evaluation and implementation of the ProMOTE intervention.

Informed by theoretical foundations and previous qualitative research, we designed the ProMOTE intervention for the home-based physically frail older adult and their caregiver (Supplement 1). The outcome is in line with other studies. Berger’s study highlights that health promotion has been shown to improve occupational performance and quality of life of older adults. The ProMOTE intervention includes sharing information with the older adult, and where relevant the informal caregiver, to promote an understanding of available options for health care. An informed person is essential to implement shared decision making [16]. Nilsen highlights that occupation-focused and occupation-based intervention using cognitive, behavioral and environmental strategies may significantly improve occupational performance in older, home-dwelling adults with physical health problems. The ProMOTE intervention study focuses on meaningful activities. Meaningful activities are daily activities tailored to the needs and preferences of the individual. The occupational therapist pursues these activities by using cognitive strategies, such as including giving information on the intervention opportunities, behavioral strategies, such as training the recommended behavior change, and environmental strategies, such as training in the use of the recommended devices and adaptations [17].

The guideline on OT for maintaining the functional performance and social participation of the home based physically frail older adults is complementary with the PRoMOTE intervention in the sense that it delivers recommendations that can be linked at each phase of the ProMOTE intervention. These recommendations support an evidence-based and client-centered approach. E.g., the ProMOTE intervention proposes that the occupational therapist uses performance-based measurement instruments to assess the older adult’s ability to perform daily activities and to participate in social life. The guideline recommends concerning operational components on this measurement among which: the occupational therapist (1) provides sufficient time to map the functional performance in the field of activities of daily living (ADL and IADL) using a standardized assessment tool (observation, interview or self-assessment tool by older adult or informal caregiver) (Grade 1 C); (2) adapts the choice of instrument to the person and their context, considering, among other things, possible floor and ceiling effects (Grade 1 C); (3) spread the testing over time if this is desirable (GPP) and (4) when interpreting the scores of ADL and IADL measurements, not only the total score is considered, but also the sub-scores (Grade 1 C) [18].

In the ProMOTE intervention, the occupational therapist collaborates with the older adult and their caregiver within an integrative care team to deliver on an evidence -based manner flexible, person-centered, integrated and proactive care. The need for a high-adherence-to-therapy OT intervention which is based on evidence-based components to optimize the occupational performance and social participation of the physically frail older adult residing at home is addressed.

This OT intervention has been developed for implementation in the Belgian primary health care context. With appropriate context adaptation, it should be transferable to other primary health care structures.

### Strengths, limits and challenges of this study

Among the strengths of this study are: (1) the establishment of theoretical underpinnings through implementation of the MRC framework for designing and evaluating complex interventions with supplemental use of the steps of the Intervention Mapping approach; (2) the evidence-based approach; (3) input from health professionals and informal caregivers regarding their insights into collaboration; (4) invoking the TIDieR checklist to improve the completeness of reporting, and (5) the piloting of the ProMOTE intervention to evaluate its feasibility, which included the inventory of barriers and facilitators of implementation.

Meanwhile, the combination of the MRC framework for designing and evaluating complex interventions with the Intervention Mapping approach has successful been implemented by one of the authors in a research on promoting empowerment for people living with dementia in nursing homes. This strategy could be considered as novel approach for other studies in future [30].

We believe that a positive result of the intervention will be the maintenance of the older adult’s functional performance in meaningful daily activities, which is a key determinant of successful and healthy aging [53–55]. The OT intervention focuses not only on the improvement of functional performance and social participation, but also on improvement of health literacy and improvement of adherence to the OT recommendations. These outcomes promote resilience, which is useful to help find solutions to new problems independently and to seek balance among daily occupations [56, 57]. Positive relationships with healthcare users are shown to be vital for therapeutic outcomes and users’ satisfaction [51].To empower people, in particular to strengthen resilience and improve therapy adherence, this study emphasizes from the outset the value of ‘use-of-self’ by fostering a relationship of trust, among others.

The interprofessional approach, which is essential for coping with the complexity of comorbidities within chronic diseases, is covered by the OT intervention [58, 59]. Case finding is described as an interprofessional fact. To facilitate adherence improvement, the intervention plan is shared with the general practitioner and the other health professionals. Interprofessional meetings are organized if it proves necessary. The OT intervention stimulates interprofessional collaboration throughout the entire therapy process, whether or not this takes place under the coordination of a care coordinator.

A limitation of this study to take under consideration is the methodological quality of the reported studies on adherence. A double-blind RCT is in general the best method of evaluating health care interventions. Differences in outcomes between groups can be confidently attributed to the difference in interventions received if contextual factors are controlled and patients are randomized to alternative comparison groups [60]. Studies on adherence are often part of complex interventions. Due to the complexity of the interfering factors, the risk of bias in complex interventions is typically high [61, 62]. For this reason, the reported studies on adherence are in general of low methodological quality.

An update of the MRC framework took place at the end of 2021 [24]. No conflicts between the framework used in this study and the updated framework were identified.

A future challenge of this study is to evaluate the ProMOTE intervention on its (cost-) effectiveness. To date, we have only piloted the intervention on feasibility aspects [34]. We did not perform a pilot-feasibility trial for evaluating limited effectiveness or a randomized controlled trial for evaluating effectiveness, therefore we did not measure the effect of the intervention. The evaluation of effectiveness can provide evidence for the decreased need for home care and the risk associated with early admission to nursing homes. The outcome of the effectiveness study should encompass the occupational performance of home-based physically frail older adults, the burden experienced by the informal caregiver of the older adults, and the level of therapy adherence among older adults participating in the ProMOTE intervention. A pragmatic RCT is a suitable design to measure the effectiveness of the ProMOTE intervention. To avoid performance bias, a cluster RCT should be considered. To verify the outcome***s***, an intention-to-treat analysis must be conducted.

Performing a cost-effectiveness study is essential to gauge the efficacy of the ProMOTE intervention in relation to the expenditure incurred for generating the outcomes. This study is imperative to advocate for increased government funding in support of occupational therapy within primary care. In this study, both a cost-effectiveness analysis and a cost utility analysis must be considered. The goal of the cost-effectiveness analysis is to provide data on the cost of the health benefits provided by the ProMOTE intervention. A cost-effectiveness analysis estimates the economic costs associated with the intervention, including both direct and indirect costs. The aim of the cost-utility analysis is to assess the impact of the ProMOTE intervention on health-related quality of life. Cost-utility is often measured using Quality-Adjusted Life Years (QALYs).A subsequent challenge is to measure the impact of an OT intervention in clinical OT practice. The impact of OT with regard to improved outcomes and lower costs is a concern of integral quality management. To measure a sustainable impact of the OT practice, the WFOT developed QUEST [52]. QUEST is a valuable tool for demonstrating the value of the OT intervention within the OT practice from seven dimensions, specifically appropriateness, sustainability, accessibility, efficiency, effectiveness, person-centeredness and safety. With these dimensions, not only the effect, but also the conditions determining the chance of success of an intervention have been mapped out. Appropriateness requires that the right OT service is delivered to the right person in the right place by the right provider, and all at the right time. Sustainability reflects the continued improvement and extension of quality OT services into the future. Accessibility refers to the ease of obtaining OT services. These seven dimensions must be made operational in terms of effect, structure and process indicators. In this way, not only the effect of the OT intervention is measured, but also the reasons for whether an effect is achieved and if implementation is expected [63].

Finally, to disseminate the intervention at national level, higher education must be involved. The training of OT students and further training of occupational therapists in clinical practice is a prerequisite for a wide implementation of the OT intervention.

### What this study adds

To the best of our knowledge, this study is the first that describes the development and content of a high-adherence-to-therapy and evidence-based evidence based OT intervention aimed at increasing functional performance and social participation of community-dwelling frail older adults.

This study provides researchers, clinicians, and decision makers transparency about the development and therefore also about the validity of the ProMOTE intervention.

Combining the MRC framework with the Intervention Mapping approach in the way we performed it, is a method that enables to further detail the intervention, especially when it concerns an intervention for complex bio-psycho-social diseases. This method may enable other researchers to take maximum account of all components that influence the development of a complex intervention.

ProMOTE is an intervention based on evidence-based components and existing of six steps, describing in detail, per step the goal, actions to be taken, the approach and communication, the resources needed, the timeframe, the involved partners and measurable quality indicators of the intervention. With these steps, both the clinical and research work field must be able to implement the ProMOTE intervention. The ProMOTE intervention describes not only ‘what’: the added value lies in the interweaving of the ‘why’ and ‘how’. By describing the ‘how’, our study makes the concept of ‘therapeutic use-of-self’ operational throughout the six steps of the ProMOTE intervention.

## Conclusion

This study transparently reports on the development process and content of a high-adherence-to-therapy and evidence-based OT intervention to optimize the functionality and social participation of the physically frail older adult living at home. The ProMOTE intervention might contribute to safely aging in place and to maintaining social participation.

The precise description of how the intervention was developed provides the opportunity to verify the validity of the development process. (Supplementary Material 1)

A further rigorous study on the effect of the ProMOTE intervention on adherence, functional performance and social participation, and of the cost-effectiveness of this intervention is strongly recommended. The outcome of an effect study may facilitate the implementation of this intervention on a national level. To understand the value of the ProMOTE intervention in terms of assessing the money spent to produce beneficial outcomes a cost-effectiveness study is desirable This study is necessary to persuade the government to consider allocating additional funds for occupational therapy in primary care. An additional cost-utility analysis is desirable to obtain insight on the impact of the ProMOTE intervention on health-related quality of life.

### Electronic supplementary material

Below is the link to the electronic supplementary material.

Supplementary Material 1

## Data Availability

All data generated or analysed during this study are included in this published article and its supplementary information file.

## Abbreviations

OT: occupational therapy
ProMOTE: promoting meaningful activities by occupational therapy in elderly
MRC: Medical Research Council
TIDieR: Template for Intervention Description and Replication
BelRAI: Belgian Resident Assessment Instrument
PI: process indicator
OI: outcome indicator
SI: structure indicator
GPP: good practice point
