# Supplementary material for: Promoting meaningful activities by occupational therapy in elderly care in Belgium: the ProMOTE intervention
Source: BMC Geriatr. 2024 Mar 20;24:275. doi: 10.1186/s12877-024-04797-6 (PMC10953191; doi:10.1186/s12877-024-04797-6)
Supplement: Supplementary file 1 — Supplementary Material 1 [file 12877_2024_4797_MOESM1_ESM.pdf]

Supplement 1: Content of the ProMOTE intervention

Goal (why)	Action (what)	Approach and communication (how)	Resources (what and how)	Collaboration (who and how)	Timeframe (when)	Quality indicator (OI: outcome indicator; PI: process indicator; SI: structure indicator)
**Case finding**						
Detecting the older adults at risk	Detection of older adults who are physically frail or at risk of physical frailty during routine daily practice of relevant health professionals and social workers<br><br>Getting to know the local health professionals	Embedding a strategy to identify cases in the daily routine care of the involved health professionals in primary care preferably supported by Computerized Decision Support System<br><br>Organizing interprofessional meetings	Clinical Frailty Scale implemented in computerized decision support system of electronic health record of all involved health professionals<br><br>If no electronic health record is in use, screening instruments such as clinical Frailty Scale or InterRAI-Pre-screener Assessment Tool or BelRAI-screener must be routinely implemented in daily practice<br><br>Using local awareness-raising work packages	Referring the detected person to the occupational therapist, and by extension to other relevant health professionals<br><br>Organizing interprofessional meetings to get acquainted with the local health professionals and to acquire more knowledge about the professional profile of each health professional	As part of the daily clinical action of the health professional<br><br>Older adults at risk must be referred at the time of detection<br><br>Yearly interprofessional boosting meetings	Presence of computerized decision support system (SI)<br>If not: screening instrument embedded in daily routine care (SI)<br><br>Referring for OT at the time of detection (PI)<br><br>Number of participants in interprofessional meetings (OI)<br><br>Number of various professionals in interprofessional meetings (OI)
**Functional assessment**						
Gaining insight into the functional abilities and expectations of the older adult–and where relevant the informal caregiver–and determinants influencing their abilities	Older adult: Assessing the functional (dis)abilities, social participation, self-perception of these abilities, personal needs and home environment<br><br>Informal caregiver: Assessing the balance of workload – workforce and personal needs	Performing the assessment in the habitat of the older person<br><br>Spreading the assessment over two sessions to reduce the load, if necessary<br><br>Using verbal and non-verbal communication strategies to build a	Older adult: Instruments to measure functional ability and social participation, personal needs and expectations (such as intake interview or COPM) and home environment<br><br>Informal caregiver: Instruments to measure burden of the informal caregiver	Inventorying the network<br><br>After consultation with the older adult themselves, exchanging information with relevant informal caregivers in a secure way, e.g. via digital shared patient records	First contact within a week after case-finding<br><br>Functional assessment can be divided over several sessions<br><br>Consultation on the results of the assessment immediately after the assessment	Availability of the assessment instruments (SI)<br><br>Availability of the results of the assessment<br>- regarding older adult: Functional performance and social participation (OI);<br>self-perception (OI);<br>home environment (OI)

	Communicating the output of the assessment to the older adult, and if appropriate to the informal caregiver<br><br>Increasing insight into the effect of the outcomes<br><br>Increasing health literacy applied to the individual context	genuine, trusting and respectful relationship with the older adult<br><br>Using techniques of Motivational Interviewing to increase the sense of ownership				- regarding informal caregiver:<br>Burden (OI)<br>personal needs (OI)<br><br>The extent to which information about the test results is provided to the older adult and/or informal caregiver (PI)<br><br>The extent to which information on causes, prognoses and overview of solutions regarding the experienced disabilities is communicated to the older adult and/or their informal caregiver (PI)
**Goal setting**						
Achieving acceptable goals for both the older adult and the occupational therapist, and where relevant the informal caregiver	Determining the shared OT goals of both the person and occupational therapist<br><br>Determining the shared goals of both informal caregiver and occupational therapist	Sharing information with the older adult, and where relevant the informal caregiver, to promote an understanding of available options for health care to aid the person in making an informed decision<br><br>Providing information on the benefits of behavior adaptations and training.<br><br>Using principles of motivational interviewing: highlighting the benefits that improving functional performance and social participation will bring to their life and to those in their context (informal caregiver, peers and family)<br>Collaborating with the older adult to identify their priorities and goals [53]	Results of OT assessment<br><br>Therapeutic use-of-self	Communicating the outcome of the assessment and goal setting with the General practitioner, and if relevant other involved health professionals<br><br>Recording the goals in shared patient records<br><br>Organizing a multidisciplinary consultation if relevant	Immediately after the functional assessment has been performed	Presence of shared decision making during goal setting (PI)<br><br>Availability of prioritized goals (OI)<br><br>Whether or not information is shared with the general practitioner

		(Tinetti et al., 2019); Listening with intent, curbing preconceptions, soliciting the individual's perspectives and validating conclusions to incorporate reciprocal trust<br><br>Implementing at least three of the goals identified by the older adult as priority goals				
**Developing intervention plan**						
Establishing a clear plan of action to achieve the goals	Determining the optimal OT intervention prescription<br><br>Prioritizing the prescribed actions	Collaborating with the older adult, and where relevant the informal caregiver, to select and prioritize the actions to achieve the goals and to plan a feasible time schedule<br><br>Collaboration with the general practitioner regarding communication of the recommendations to the older adult and/or their informal caregiver<br><br>Encouraging the older adult to seek help and encouragement from their context	Results of goal setting<br><br>Activities that are meaningful to the older adult	Recording the intervention plan in the shared patient records<br><br>Providing possibility to organize interprofessional meetings where necessary, e.g. to increase therapy adherence<br><br>Providing possibility of care coordination or case management	Following the goal setting	Availability of an intervention plan (OI)<br><br>The extent to which actions are selected in collaboration with the older adult (PI)<br><br>The extent to which information is shared with the General practitioner (PI)
**Implementing and monitoring intervention plan**						
Pursuit of success in achieving the goals and gaining insight into the progress	Implementing the prescribed actions in phases<br><br>Detecting new and unexplored limitations	Dividing the actions and recommendations over several sessions<br><br>Number of sessions can vary depending on various factors, such as the complexity of the case and the level of health literacy	Habitat of older person<br><br>Intervention plan<br><br>Therapeutic use-of-self	Recording status praesens of intervention in the shared patient records	Reducing the time between the assessment and the actual implementation of recommendations as much as possible	The extent to which the prescribed actions are implemented in phases (PI)<br><br>The extent to which the goals are reached after implementation of the intervention plan (OI)<br><br>The extent to which the prioritization of goals and

		Adjusting where necessary and in shared decision-making procedures<br><br>Continuously communicating the actions associated with the goals to the older adult, and if relevant the informal caregiver<br><br>Reminding the older adult to seek help and encouragement from their context (informal caregiver, peers and family)<br><br>Naming objectives that are achieved				actions was considered during implementation (PI)
**Evaluating**						
Determining whether treatment can be finalized or must be adjusted	Older adult: Interviewing the self-perception of functional (dis)abilities and social participation<br><br>RE-assessment of the home environment<br><br>Informal caregiver: assessing the perceived balance of workload – workforce and personal needs<br><br>Writing a final report	Communicating the outcome of the evaluation with the older adult, and where relevant the informal caregiver<br><br>Using open-ended questions	Using standardized OT assessment instruments where relevant	Sharing the final report with the general practitioner and other relevant health professionals	Evaluation takes place during the intervention process (to adjust if necessary) and at the end of the intervention	Whether or not the final report is available (OI)<br><br>Whether or not the final report is shared with the interprofessional team (PI)
